# Analysis of reasons for loss to follow up in a prospective study in Chandigarh, India and impact from telecom changes

**DOI:** 10.1186/s13104-021-05837-9

**Published:** 2021-11-18

**Authors:** Joseph L. Mathew, Pooja N. Patel, Abram L. Wagner, Vanita Suri, Bhavneet Bharti, Bradley F. Carlson, Matthew L. Boulton

**Affiliations:** 1 Advanced Pediatrics Center, PGIMER, Chandigarh, 160 012 India; 2 Department of Epidemiology, School of Public Health, University of Michigan, 1415 Washington Heights, Ann Arbor, MI 48109 USA; 3 Department of Obstetrics and Gynecology, PGIMER, Chandigarh, 160 012 India; 4 Department of Internal Medicine, Division of Infectious Disease, University of Michigan Medical School, 1500 East Medical Center Drive, Ann Arbor, MI 48109 USA

**Keywords:** Loss to follow-up, mHealth, Telecommunications

## Abstract

**Objective:**

Mobile phones are used in research studies, to enroll and follow-up participants, collect data, and implement mHealth initiatives. We conducted a longitudinal study in a birth cohort, where infants were required to make four scheduled visits by 12 months of age. Families of those failing to attend scheduled follow-up visits, were contacted telephonically to ascertain the reasons, which were categorized as: not interested to continue participating, migrated, phone disconnected due to telecom change, or other reason.

**Results:**

A total of 413 mother-infant dyads were enrolled. The overall attrition was 56%, with majority occurring at the first follow-up visit. This temporally coincided with a telecom service provider announcing strong incentives to switch providers. Attrition monotonically decreased at subsequent visits. The reasons were: moved away (13%), no longer interested (8%), phone disconnected (7%), and multiple other reasons (28%), the majority of whom had unreachable phones. Those who remained in the study and those lost to follow-up were similar on most demographic variables. Among common reasons for attrition in cohort studies, we experienced a new dimension introduced by telecom changes. These findings underscore the need to consider unexpected reasons for attrition in longitudinal studies, and design more robust methods to follow-up participants.

## Introduction

Longitudinal epidemiological studies have been used for decades, making key contributions to understanding the risk factors [1] and etiology of various diseases [2, 3]. The evidence base in nutrition [4], cardiovascular disease [5], and host responses to infection [6] and many other health conditions, depends upon longitudinal studies [7, 8].

Currently, there is increasing interest in the impact of mHealth (mobile health) initiatives on various health outcomes [9, 10], particularly in low- and middle-income countries (LMICs) where there has been unprecedented growth of mobile phone networks [11]. Mobile phones are being used in longitudinal studies for contact with study participants, data collection, and various mHealth interventions. Such use of mobile phones is even a focus of funding agencies like the US National Institutes of Health [12] and the Gates Foundation [13].

However, the inherent need to repeatedly contact participants during a longitudinal study is challenged by attrition (i.e., loss to follow-up). Differential loss to follow-up can bias study results and also impact the ability to generalize the findings, if there are substantial differences between those lost to follow-up and those retained in the study. Prospective studies relying on mobile phones encounter problems if participant(s) or telecom provider(s) disconnect phones or alter coverage plans, during the course of the study. Problems resulting from multiple phones, disconnected phones, or switching telecom providers are likely exacerbated in LMICs where there may be limitations in reliable additional mechanisms for contact [14].

In a prospective birth cohort study in India, investigating the decay of maternal antibodies to measles among infants, we collected data at infants’ birth and at three, six, nine, and twelve months of age. During the study period, a telecom provider offered strong incentives for individuals to switch their mobile phone provider [15, 16], due to which phone numbers recorded for the study were no longer working. In this study, we examined the reasons for participant attrition, and also compared demographic characteristics between those lost to follow-up and those remaining in the study.

## Main text

### Methods

#### Study population

We randomly selected mother-infant dyads at birth, from 30 Anganwadi served communities, representative of the population residing in rural, urban, and resettlement colonies of Chandigarh, India. Data collection occurred during January 2017–October 2018.

Study staff first identified potential participants i.e., pregnant women, from Anganwadi records. For inclusion into the study, they had to be residing in Chandigarh, India at the time of delivery, and intending to reside in Chandigarh for at least a year after delivery. Study staff contacted each individual up to four times prior to the delivery date to invite their participation. If potential participants declined to participate or Study staff were not able to reach them, the next eligible individual from the enrollment log was contacted to participate in the study.

Enrolment of pregnant women was done at delivery in the hospital labour room. The following were excluded from enrolment: those with an acute febrile illness, those with known hemophilia or other blood dyscrasias characterized by potential for excessive bleeding, or those with a health condition necessitating immune-suppression medication. Infants born from participants were enrolled immediately after delivery.

Each enrolled infant underwent four scheduled follow-up visits at the ages of 3, 6, 9, and 12 months. Visits were scheduled during routine working hours, and mentioned on the Study records provided to the family. In addition, Study staff contacted the family telephonically at least 48 h prior to the scheduled visit, to remind them about the follow-up visit.

#### Derived variables

The main outcome in this study was being ‘lost to follow-up’. If caregivers of infants declined to follow-up during telephonic reminders, or failed to follow-up despite reminders, or could not be contacted prior to, or after the scheduled visit date elapsed, the Study staff recorded the reasons for loss to follow-up. The major categories were: (1) no longer interested to participate in the research study, (2) moved away from Chandigarh, (3) phone was disconnected (i.e., the number was no longer operational), or (4) other reasons.

The predominant independent variables were infant’s sex, mother’s age (trichotomized as 18–24 years, 25–29 years, and 30–42 years), caste [scheduled casted/scheduled tribe, other backward caste (OBC), or other], religion (dichotomized as Hindu or not), and monthly income (< 10,000 INR, 10,000–24,999 INR, 25,000–49,999 INR and  ≥  50,000 INR, which correspond to  <  $133, $133–$333, $334–$666 and  >  $666, respectively per current exchange rates). We also considered when the drop-out occurred, as the study check points were at birth, and age of three, six, nine and twelve months.

#### Statistical analysis

We summarized the number of individuals lost to follow-up, by reason. We ran two Rao-Scott chi-square tests to compare demographic information for those who were lost to follow-up versus those who were not. Reasons for loss to follow-up were also depicted graphically across study check points. The chi-square tests accounted for the survey design (clustering at Anganwadi centers). A p value  <  0.05 was considered significant. Analyses were processed in SAS version 9.4 (SAS Institute, Cary, NC).

#### Ethical approval

This study was approved by the University of Michigan Health Sciences and Behavioral Sciences Institutional Review Board (HUM00104905), the Institutional Ethics Committee of the Postgraduate Institute of Medical Education and Research, Chandigarh, and the Health Ministry Screening Committee of the Government of India. Enrolled mothers provided written, informed consent to participate. Either parent provided written, informed consent for enrolment of their infants.

## Results

**Fig. 1:**
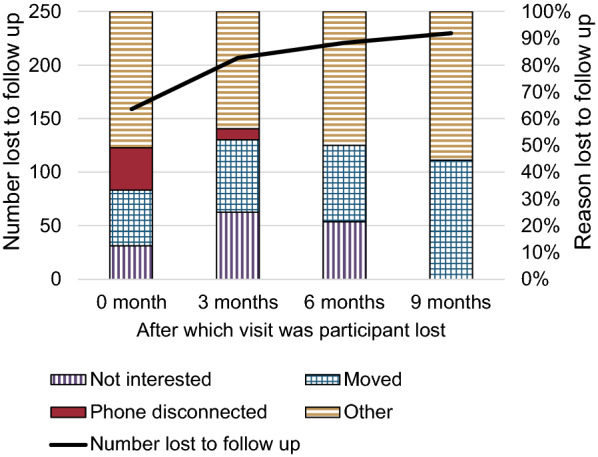
Loss to follow up over the course of a 1 year time period in Chandigarh, India. Other reason” included 107 participants who were not reachable, 2 who had died, and 5 who were out of town on the follow-up date

Overall, 1810 mothers were screened and 413 mother-infant dyads were enrolled. A little over half (230, 56%) were lost to follow-up during the 12-month follow-up period. Most attrition (159 of 230, 69%), occurred at the first 3-month study visit, with decreasing amounts subsequently; 48 (21%) after three months of age, 14 (6%) after six months of age, and (4%) after nine months of age (Fig. 1). Of those who were lost to follow-up, the majority (114 of 230, 28%) were lost due to “other reason” which comprised 107 individuals who could not be contacted at all (i.e., phone was persistently unreachable), two infants who had died, and five who were out of town on the follow-up date (Table 1). Approximately 13% did not continue in the study because they moved from Chandigarh, and 8% were not interested in continuing. Those who were lost to follow-up due to a disconnected phone constituted 7%.

**Table 1:** Demographic characteristics of participants in the cohort and those lost to follow up

	Whole cohort (col. %)	Retained (row %)	P^a^	Lost to follow up (n = 230, 56%)			
				Not interested (row %)	Moved away (row %)	Phone disconnected (row %)	Other reason^b^ (row %)
Overall	413	183 (44%)		35 (8%)	54 (13%)	27 (7%)	114 (28%)
Child’s sex			0.402				
Male	202 (48%)	95 (47%)		17 (8%)	25 (12%)	12 (6%)	53 (26%)
Female	211 (50%)	88 (42%)		18 (9%)	29 (14%)	15 (7%)	61 (29%)
Mother’s age			0.749				
18–24 years	105 (25%)	49 (47%)		7 (7%)	21 (20%)	3 (3%)	25 (24%)
25–29 years	181 (43%)	81 (45%)		16 (9%)	26 (14%)	12 (7%)	46 (25%)
30–42 years	127 (30%)	53 (42%)		12 (9%)	7 (6%)	12 (9%)	43 (34%)
Caste			**0.015**				
Scheduled caste/scheduled tribe	85 (20%)	46 (54%)		9 (11%)	6 (7%)	4 (5%)	20 (24%)
Other backward caste	54 (13%)	28 (52%)		2 (4%)	4 (7%)	4 (7%)	16 (30%)
Other	274 (65%)	109 (40%)		24 (9%)	44 (16%)	19 (7%)	78 (28%)
Religion			0.246				
Not Hindu	73 (17%)	37 (51%)		3 (4%)	3 (4%)	3 (4%)	27 (37%)
Hindu	340 (81%)	146 (43%)		32 (9%)	51 (15%)	24 (7%)	87 (26%)
Monthly income			0.563				
< 10,000 INR	186 (44%)	89 (48%)		11 (6%)	24 (13%)	11 (6%)	51 (27%)
10,000–24,999 INR	121 (29%)	52 (43%)		9 (7%)	19 (16%)	8 (7%)	33 (27%)
25,000–49,000 INR	82 (19%)	34 (41%)		10 (12%)	9 (11%)	7 (9%)	22 (27%)
≥ 50,000 INR	24 (6%)	8 (33%)		5 (21%)	2 (8%)	1 (4%)	8 (33%)

^a^Rao-Scott chi-square test comparing those retained versus those lost to follow up

^b^“Other reason” included 107 participants who were not reachable, 2 who had died, and 5 who were out of town on the follow-up date

Infants retained in the study and those who were lost to follow-up, did not differ significantly on most demographic variables (infant’s sex, mother’s age, religion, and monthly income). Caste was associated (p  = 0.015) with loss to follow-up; attrition occurred among 60% of participants categorized as “Other”, 46% among scheduled caste/scheduled tribe and 48% among OBC, respectively. Both mother’s age and monthly income had an inverse relationship with the percentage of participants retained in the study, although neither was statistically significant.

## Discussion

This study showed that among various reasons for participant attrition in prospective community-based cohort studies, ‘telecom changes’ introduced a new dimension that made it difficult (or impossible) to track participants. Since this can impact other similar studies, we suggest practical ways to manage the problem, within limited resources.

Overall, we experienced significant attrition with the majority occurring during the first follow-up visit. Although  <  10% attrition was definitely attributable to telecom changes, (identified through automated messages that the service was unavailable), this is likely a conservative estimate. A significant proportion of those who could not be contacted at all, were also likely due to telecom changes, since most were unreachable on multiple attempts (with automated recordings persistently stating that the subscriber was not reachable).

Telecom-related issues are important given the increasing use of mobile phones for mHealth initiatives. India has the second largest telecommunications network with over 1 billion users [17], and the industry faces high customer churn and switching of telecom providers [18]. One study found that call rates were the most important factor in switching mobile service providers and almost half the respondents were likely to switch to another service provider [19] often preferring to opt for a new phone number rather than porting existing number to the new provider.

Other factors, including gender norms and socio-economic status, also intersect to influence mobile phone use [20]. Previous research identified an association between lower socioeconomic status and increased loss to follow-up [21]. We found the opposite relationship; those of higher socioeconomic status being more likely to be lost to follow-up. It is possible that certain groups (with more disposable income, or of a certain caste) might be attuned to changes in telecom services.

Loss to follow-up has significant implications for longitudinal studies and can lead to biased estimates [22] and limited generalizability. For studies relying on phones to contact participants or deliver mHealth interventions, it may be of value to provide study participants with a mobile device, or to provide funds to cover mobile plan costs. Study staff could also collect multiple phone numbers at the time of enrollment; one for the primary study participant and another of a family member. Such strategies, if appropriate in the cultural context, could potentially reduce the loss to follow-up due to telecom related issues. Researchers could also assess the feasibility of using social media platforms including apps like Whatsapp (that are very popular in India).

We did not use (automated or other) text messaging to contact participants. First, we did not have the infrastructure to send out automated text message reminders. Second, many service providers provide pre-paid usage plans that provide unlimited (outgoing and incoming) calling but only a limited number of free-of-charge text messages. Third, telecom service providers regularly send advertising text messages to users, resulting in them being ignored. Fourth, older non smartphone handsets have limited inbox capacities, and fail to receive text messages unless the inbox is periodically emptied.

Previous studies have shown that increasing funds for study staff to track down individuals can decrease attrition [23]. In addition, loss to follow up can also be reduced by implementing a comprehensive protocol including an electronic database, training in cultural/linguistic competency, reinforcing study importance, and home assessment visits [24]. Reinforcing study importance could be particularly important in a study like this one if, for example, individuals do not believe vaccination to be important or measles disease to be serious.

The findings of this study can serve to inform the design of longitudinal studies in LMICs. The use of mHealth modalities are likely to increase as mobile phone access increases. Therefore addressing this issue will become even more important over time. This is especially true as major funders of global research, like the Gates Foundation, often reward proposals using technology and technology-driven programs to conduct studies and carry out interventions. The challenges may compound with time as more carriers and therefore more options for customers become available particularly in LMICs where many of these studies are conducted.

## Conclusions

This study showed that, among multiple causes for participant attrition in prospective community-based cohort studies, a new dimension was introduced by ‘telecom changes’ that made it difficult (or impossible) to track participants. Practical ways to manage this problem without intensive investments in human and material resources, could be by recording multiple contact details for each participant, exploring the use of instant messaging platforms (or social media), and maintaining more regular contact with participants.

## Limitations

The study has several limitations. First is the possible misclassification of the outcome. Despite thorough training, Study staff may have recorded the primary reason for loss to follow-up in a varying manner. Second, we could not ascertain whether those who were persistently “not reachable” to study calls, had disconnected phone-service due to telecom changes. Those with automated recorded messages stating that “the subscriber does not exist” or “this number does not exist” were easier to classify, compared to those without automated recordings. Third, the study was conducted in a single Indian city in a region that is culturally, socially, and geographically diverse, limiting its overall ability to be generalized. Last, we had a relatively small sample size, limiting confidence to detect significant effects or identify specific groups that are overall more likely to be lost to follow-up.

## Data Availability

Data and material used in this study are available with the authors on request. Researchers requiring additional data are invited to contact the Authors.

## Abbreviations

LMIC: Low- or middle- income country
mHealth: Mobile health
OBC: Other backwards caste
